# Study on machine learning of molar incisor hypomineralization in an endemic fluorosis region in central China

**DOI:** 10.3389/fphys.2023.1088703

**Published:** 2023-03-15

**Authors:** Yimeng Zhang, Yu Wang, Zhaoxin Zhang, Yuqi Wang, Jie Jia

**Affiliations:** ^1^ Henan University School of Stomatology, Kaifeng, China; ^2^ The Key Laboratory of Clinical Resources Translation, Henan University, Kaifeng, China; ^3^ The First Affiliated Hospital of Henan University, Kaifeng, China

**Keywords:** molar incisor hypomineralization (MIH), prevalence, dental fluorosis (DF), dental caries, machine learning (ML)

## Abstract

**Objectives:** The aim of the present study was to develop a machine learning model to predict the risk of molar incisor hypomineralization (MIH) and to identify factors associated with MIH in an endemic fluorosis region in central China.

**Methods:** A cross-sectional study was conducted with 1,568 schoolchildren from selected regions. The clinical examination included an investigation of MIH based on the European Academy of Paediatric Dentistry (EAPD) criteria. In this study, supervised machine learning (e.g., logistic regression) and correlation analysis (e.g., Spearman correlation analysis) were used for classification and prediction.

**Results:** The overall prevalence of MIH was 13.7%. The nomograph showed that non-dental fluorosis (DF) had a considerable influence on the early occurrence of MIH and that this influence became weaker as DF severity increased. We examined the association between MIH and DF and found that DF had a protective correlation with MIH; the protective effect became stronger as DF severity increased. Furthermore, children with defective enamel were more likely to experience caries, and dental caries were positively correlated with MIH (OR = 1.843; 95% CI: 1.260–2.694). However, gender, oral hygiene, and exposure to poor-quality shallow underground water did not increase the likelihood of developing MIH.

**Conclusions:** DF should be considered a protective factor within the multifactorial etiology of MIH.

## 1 Introduction

The European Academy of Paediatric Dentistry (EAPD) defines molar incisor hypomineralization (MIH) as enamel mineralization defects in one to four permanent first molars, with or without the involvement of the permanent incisors (Weerheijm et al., 2003). MIH-affected teeth clinically display demarcated opacity on the occlusal or buccal surfaces of the crowns (da Costa-Silva et al., 2010; Jeremias et al., 2013). MIH may be confused with dental fluorosis (DF), which shows diffuse opacity when the same teeth are affected. To date, researchers have performed many studies on the prevalence of MIH, but the study of MIH in endemic fluorosis regions is very limited.

The correlation between MIH and DF remains unclear. Studies have indicated that the presence of naturally fluoridated waters does not increase the incidence of MIH (Balmer et al., 2012; Schmalfuss et al., 2016); however, the severity of MIH is likely to be associated with DF (Fernandes et al., 2021). Significantly, the prevalence rate of MIH was lower in fluoridated areas of Northern England than in non-fluoridated areas (Balmer et al., 2012). A similar situation existed in Brazil, where a significant negative association between MIH and DF at the tooth level has been observed (Duarte et al., 2021). Therefore, it is important to study the relationship between MIH and DF, as well as the severity of MIH and DF.

Recently, machine learning methods have been used to predict a variety of diseases. Machine learning methods may be used to overcome some limitations of current analytical approaches and to find associations by applying computer algorithms to large datasets with numerous, multidimensional variables, capturing high-dimensional relationships among clinical features to obtain data-driven outcomes (Schwalbe and Wahl, 2020). Thus, we sought to develop a machine learning-based risk stratification model to explore the risk of MIH in an endemic fluorosis region in central China.

The aim of this study was to determine the prevalence of MIH and to predict its occurrence by utilizing machine learning and to explore the association between MIH and dental fluorosis in children living in an endemic fluorosis region in central China.

## 2 Material and methods

### 2.1 Ethical considerations and sample

The present study was performed with the approval of the Medical Ethics Committee of The First Affiliated Hospital of Henan University (2019LCSY-002). Signed informed consent was obtained from the caregivers and children prior to their participation in the study.

A cross-sectional study was conducted from April to June 2021 with a representative sample of schoolchildren aged 8 and 10 years in Lankao County, which is located on the eastern boundary of Henan Province. This county has endemic fluorosis, with fluoride concentrations ranging from 1.22 to 3.90 mg/L (Wang et al., 2008; Wang and Cao, 2013), which exceed the standard for drinking water quality in China (1.0 mg/L; GB5749-2006).

The formula for calculating a minimum number of randomly selected children was as follows: sample size (n) = [Z^2^×P(1-P)]/d^2^, where Z is the statistical level of confidence for a 95% confidence interval (CI; Z = 1.96), P is the expected prevalence, and d is the precision (Naing et al., 2006). Recent studies have revealed that the global average prevalence of MIH is 12.9% (11.7%–14.3%) (Schwendicke et al., 2018). According to the formula, this study required 169 participants. Schools were selected randomly according to the number of schools in each town, and a stratified sample of pupils was selected from each school according to the total number of pupils in the school. The inclusion criteria were as follows: residents of both sexes, aged 8–10 years, born and raised locally, with all four permanent first molars and incisors fully erupted. The exclusion criteria were as follows: having no erupted permanent first molar and incisor, undergoing fixed orthodontic treatment with brackets or bands on permanent first molars, and defects less than 1 mm in diameter.

### 2.2 Training and calibration of examiners

The European Academy of Paediatric Dentistry (EAPD) (Weerheijm et al., 2003) criteria for MIH were used in this study. Calibration exercises were conducted among three MIH investigators using clinical photographs of 26 patients. The tooth defects of patients covered all the degrees of MIH and other enamel defects, such as dental fluorosis, hypoplasia, and amelogenesis. The validity of using clinical photographs to study enamel defects was previously confirmed by Sabieha and Rock (1998), Wong et al. (2006), and Yi et al. (2021). After 1 month of training, three examiners were able to correctly diagnose all cases independently. Cohen’s kappa coefficients for inter- and intra-rater reliability were 0.92 and 0.89 for dental fluorosis, 0.86 and 0.75 for dental caries, and 0.65 and 0.77 for MIH, respectively. Furthermore, during this month, three investigators examined 10 enamel defect patients who visited the Department of Stomatology, which guaranteed that the three investigators were familiar with the diagnosis and management of children with MIH.

### 2.3 Dental examination

Participants were advised to brush their teeth before the exam, and the teeth stayed slightly wet during the process of inspection. The items to be prepared included a simple dental chair with a dental light source (DYNAMIC, China), disposable oral treatment plates, disposable gloves, and cotton balls.

MIH: Clinical examinations were carried out by unified trained specialist dentists and comprised examination for developmental enamel defects and dental caries using the EAPD criteria (Weerheijm et al., 2003). To guarantee between-examiner reproducibility, the examinations were performed jointly by two dentists. A specially designed chart was used to record sex, the year of birth, the presence of MIH, the number of affected incisors and molars, and the maximum degree of severity. Severity was quantified according to clinical appearance (Ghanim et al., 2017) and was classed as 1) mild, including white and yellow demarcated opacities; or 2) severe, including posteruptive enamel breakdown (PEB), atypical restoration, atypical carious lesions, and missing due to MIH.

Dental fluorosis: The Thysltrup–Fejerskov (TF) criteria were used to determine the occurrence of dental fluorosis with an ordinal scale from 0 to 9 (Thylstrup and Fejerskov, 1978). Based on the loss of structure, teeth with a TF of 0 were classified as normal, those with a TF of 1-4 were classified as mild, and those with a TF greater than 5 were classified as severe.

Dental caries: Clinical dental caries (manifest caries) were recorded as decayed, missing, or filled teeth (DMF). Decay was defined as visible tooth substance loss without the characteristics of developmental defects, pits, or fissures.

Oral hygiene status: Oral hygiene was recorded using the simplified oral hygiene index (OHI-S) described by Greene and Vermillion (1964). The level of oral hygiene was evaluated according to the debris index and was classed as fair (0) or poor (1).

### 2.4 Databases and data preprocessing

To develop the machine learning models, we used a derivation cohort of children who met the inclusion criteria. The raw dataset contained the study subjects’ demographics and the results of the comprehensive oral examination. Initially, the dataset used for preprocessing and classification was collected. The main characteristics of this dataset included MIH, MIH severity, DF, DF severity, dental decay, oral hygiene, water quality, and sex.

### 2.5 Machine learning methods and statistical validation strategies

**FIGURE 1:**
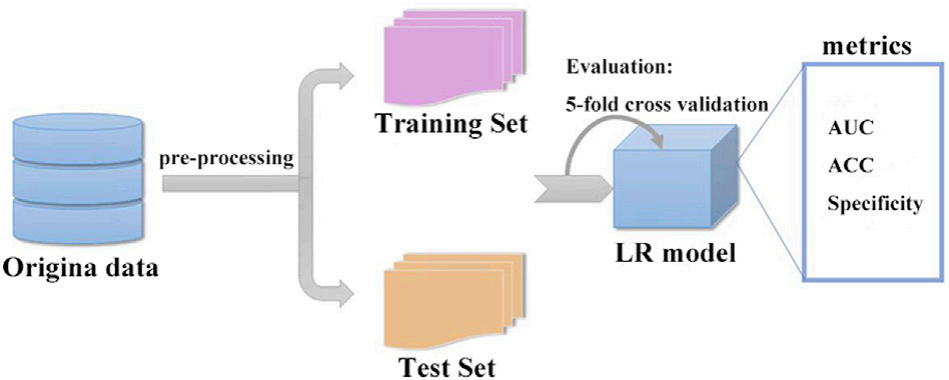
Structure of the supervised machine learning algorithm, which can describe this network working principle satisfactorily. The derivation cohort was randomly split into two datasets: a training set (70%) and an internal validation set (30%). After training and validation, we successfully constructed a logistic regression model (LR model).

The derivation cohort was randomly split into two datasets: a training cohort (70%) used to train the machine learning model and tune the parameters, and an internal validation cohort (30%) used to test the developed model on unseen data and to fine-tune the hyperparameters. For training, the original data space was balanced by oversampling using SMOTE. It worked by adding small samples from the data space to diminish the biased behavior of imbalanced data, thus changing the size of the training data space. In this study, supervised machine learning (e.g., logistic regression) and correlation analysis (e.g., Spearman correlation analysis) were used for classification and prediction. Once the number of models was considered for this particular study, we used accuracy (ACC), specificity (SPEC), and the ROC curve and area under the curve (AUC) to validate the prediction performance for binary classes (Figure 1).

### 2.6 Statistical analysis

The completed examination records were analyzed by the SPSS Statistics 22.0 program (IBM SPSS, Chicago, IL, United States of America), Python (version 3.9.7), and R (version 4.1.3). The presence of MIH was considered a dependent variable. Dental caries and dental fluorosis were considered independent variables. Confidence intervals of 95% were calculated for prevalence. Chi-squared tests and Fisher’s exact tests were used for comparisons and correlations. Logistic regression was conducted to analyze factors that could affect MIH. Significance was set at a *p*-value of < 0.05.

## 3 Results

### 3.1 Distribution characteristics of MIH in the population

A total of 1,586 children were invited to participate. Of these, 18 were not in accordance with the inclusion criteria. The clinical characteristics and demographics of the study population are shown in Table 1. The dataset consisted of 1,568 samples with two types: MIH (215, 13.7%) and non-MIH. This record included 755 male and 813 female. Patients with MIH consisted of a greater proportion of males—;109 (14.4%) were male and 106 (13.0%) were female, —but the difference was not statistically significant. For permanent teeth, caries activity in Lankao City was low, at 5.6% in only permanent teeth and 14.2% in permanent and deciduous teeth. As expected, the percentage of dental fluorosis was as high as 54.8% (Table 1). There was a higher percentage of children with poor oral hygiene in this region (91.5%).

**TABLE 1:** Baseline features of the included cohorts.

Characteristic	Total	MIH group	Normal group	χ^2^	P
	(n = 1,568), n (%)	(n = 215), n (%)	(n = 1,353), n (%)		
Sex				0.648	0.421
Male	755 (48.15)	109 (50.7)	646 (47.75)		
Female	813 (51.85)	106 (49.3)	707 (52.25)		

Water quality				2.881	0.090
Normal quality	769 (49.04)	117 (54.42)	652 (48.19)		
Lower quality	799 (50.96)	98 (45.58)	701 (51.81)		

MIH degree					
Mild		85 (39.53)			
Severe		130 (60.47)			

Dental fluorosis				58.267	<0.001
Normal	709 (45.22)	148 (68.84)	561 (41.46)		
Mild	778 (49.62)	65 (30.23)	713 (52.7)		
Severe	81 (5.16)	2 (0.93)	79 (5.84)		

Dental decay				129.371	<0.001
normal	406 (25.89)	37 (17.21)	369 (27.27)		
Only permanent teeth	88 (5.61)	32 (14.88)	56 (4.14)		
Only deciduous teeth	852 (54.34)	74 (34.42)	778 (57.5)		
Permanent teeth and deciduous teeth	222 (14.16)	72 (33.49)	150 (11.09)		

Oral hygiene				0.106	0.745
Fair	133 (8.48)	17 (7.91)	116 (8.57)		
Poor	1,435 (91.52)	198 (92.09)	1,237 (91.43)		

**FIGURE 2:**
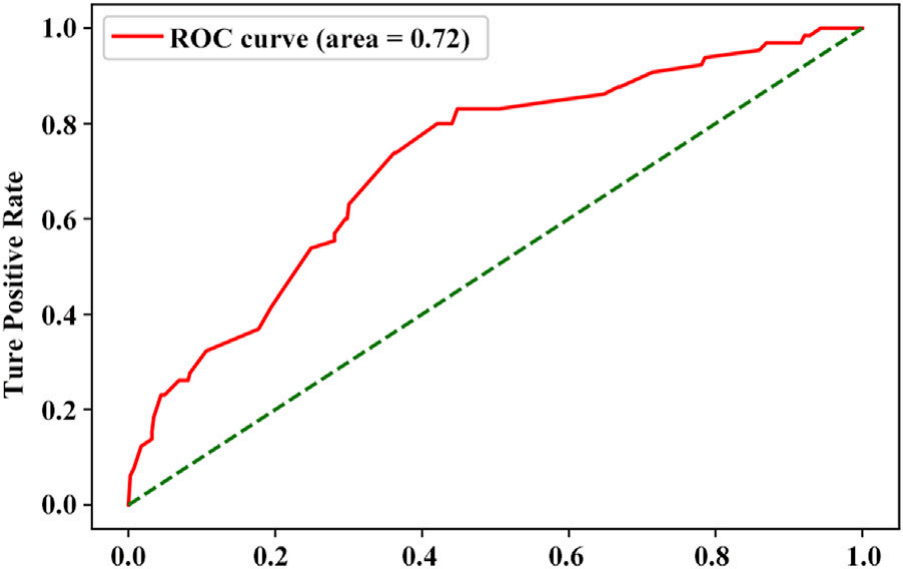
ROC curve for MIH in participants. ROC, receiver operating characteristic curve.

A supervised machine learning algorithm (e.g., logistic regression) was used to check for the occurrence of MIH. A total of 1,568 samples were analyzed, and six variables were included. During data preprocessing, the diagnosis of non-MIH or MIH was encoded using a binary encoder as 0 and 1, respectively. The ROC curve analysis was significant for this model, displaying an area under the curve of 0.72 (Figure 2). This model showed an accuracy of 70% and a specificity of 72%.

### 3.2 Distribution of MIH severity

**FIGURE 3:**
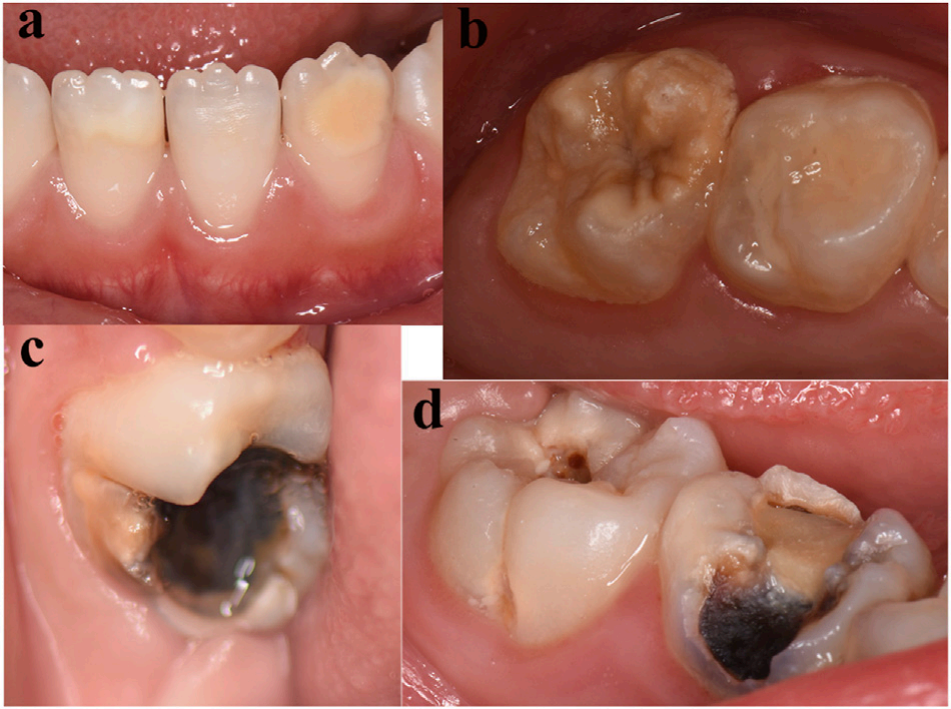
Phenotype of MIH. The majority of hypomineralized permanent teeth exhibited white **(A)** and yellow **(B)** demarcated lesions. **(C)** Atypical carious lesions associated with demarcated opacities in the first permanent molar. **(D)** Typical caries in the first permanent molars and atypical restorations in the primary molars.

The typical clinical phenotype of MIH is shown in Figure 3, including mild and severe types. With respect to the distribution of MIH severity among affected index teeth, demarcated opacities comprised the predominant type of defects (Table 2).

**TABLE 2:** Severity of MIH (number of teeth).

	Mild type	Severe type			
	**Demarcated opacity**	**Post-eruptive enamel breakdown**	**Atypical restoration**	**Atypical caries and missing duo to MIH**	**Total**
	n %	n %	n %	n %	n %
Total	285 (59.1)	38 (7.9)	7 (1.5)	152 (31.5)	482 (100)

### 3.3 Distribution of caries

The examined population presented a mixed dentition, and data from primary and permanent dentitions were presented separately (18,714 deciduous and 18,918 permanent teeth) (Table 3).

**TABLE 3:** Incidence of caries (number of teeth).

	Decay	Missing	Filling	Normal	Total
	n (%)	n (%)	n (%)	n (%)	n (%)
Permanent teeth	513 (2.71)	7 (0.04)	35 (0.19)	18,363 (97.06)	18,918 (100)
Deciduous teeth	3,773 (20.16)	21 (0.11)	52 (0.28)	14,868 (79.45)	18,714 (100)

### 3.4 Nomogram development and validation

**FIGURE 4:**
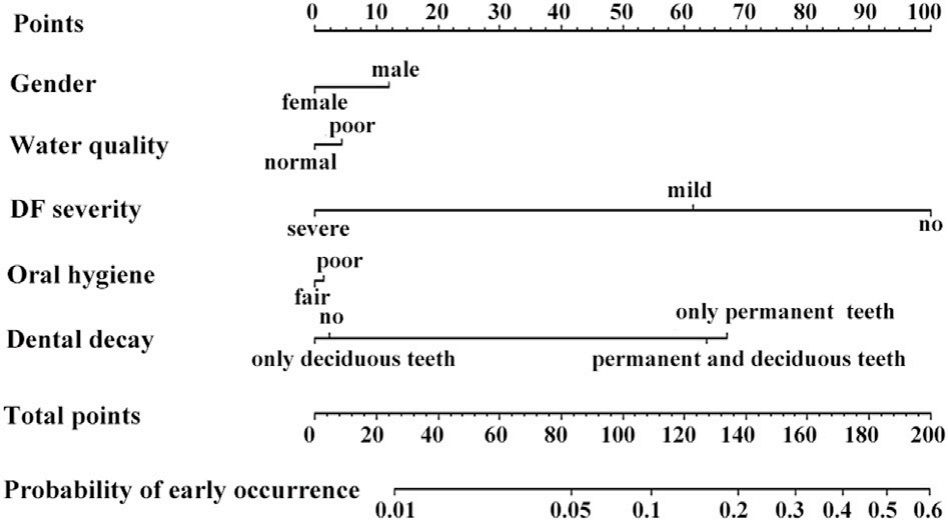
Nomograms to predict the probability of MIH occurrence. Non-DF had a significant influence on MIH occurrence, which decreased with increasing DF severity. Dental decay had a certain effect on it. However, exposure to low-quality shallow underground water did not increase the likelihood of developing MIH.

Based on a supervised machine learning algorithm, we constructed nomograms for predicting the occurrence of MIH, as shown in Figure 4. Non-DF had a great influence on the early occurrence of MIH. Significantly, the more severe the DF, the lower the occurrence.

Dental decay of permanent teeth was related to the occurrence of MIH. However, exposure to poor-quality shallow underground water did not increase the likelihood of developing MIH. Gender and oral hygiene had no influence on the occurrence.

### 3.5 Factors associated with MIH

**FIGURE 5:**
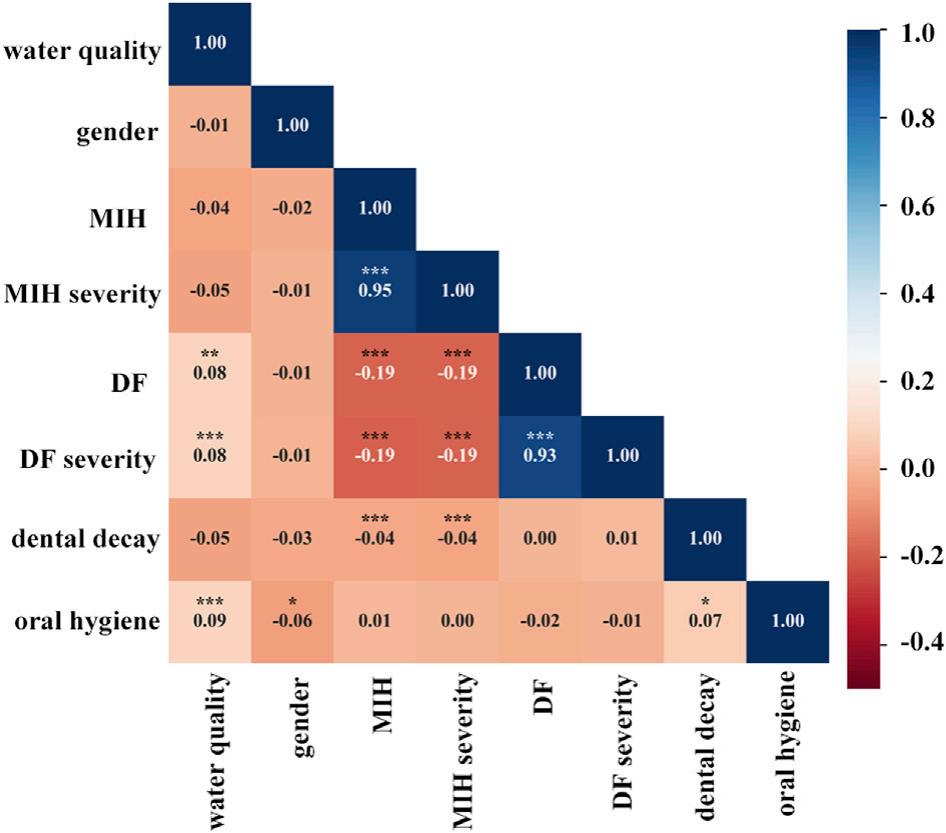
Correlation heatmap of clinical characteristics using Spearman correlations. Heatmap shows the positive (red) or negative (blue) correlations of all parameters, with color intensity reflecting the strength of the correlation (−0.4 to +1). MIH and MIH severity were negatively correlated with DF and DF severity and also had negligible associations with dental caries. *p < 0.05; **p < 0.01; ***p < 0.001.

A correlation heatmap of all parameters was generated using Spearman correlation coefficients. Figure 5 illustrates the correlation heatmaps of the core data features. MIH and MIH severity were negatively correlated with DF and DF severity. As in the logistic regression analysis, DF had a protective correlation with MIH, and the protective effect became stronger as DF severity increased. However, dental caries were positively correlated with MIH (OR = 1.843; 95% CI: 1.260–2.694) (Table 4), which was inconsistent with the heatmap. The heatmap showed a negligible correlation between MIH and dental caries.

**TABLE 4:** Logistic regression analysis of the associations between the variable of interest and MIH.

Variable	*p*-value	OR (95%CI)
Drinking water quality		
Normal quality		1
Poor quality	0.396	0.880 (0.654–1.183)

Dental caries		
Absent		1
Present	0.002	1.843 (1.260–2.694)

Dental fluorosis degree		
Absent		1
Mild	<0.001	0.349 (0.255–0.478)
Severe	0.001	0.091 (0.022–0.375)

Both the correlation heatmap and logistic regression analysis showed that the quality of underground water was not robustly correlated with MIH or MIH severity. Not surprisingly, the quality of underground water was correlated with DF and DF severity. A similar correlation was found between dental decay and oral hygiene.

**FIGURE 6:**
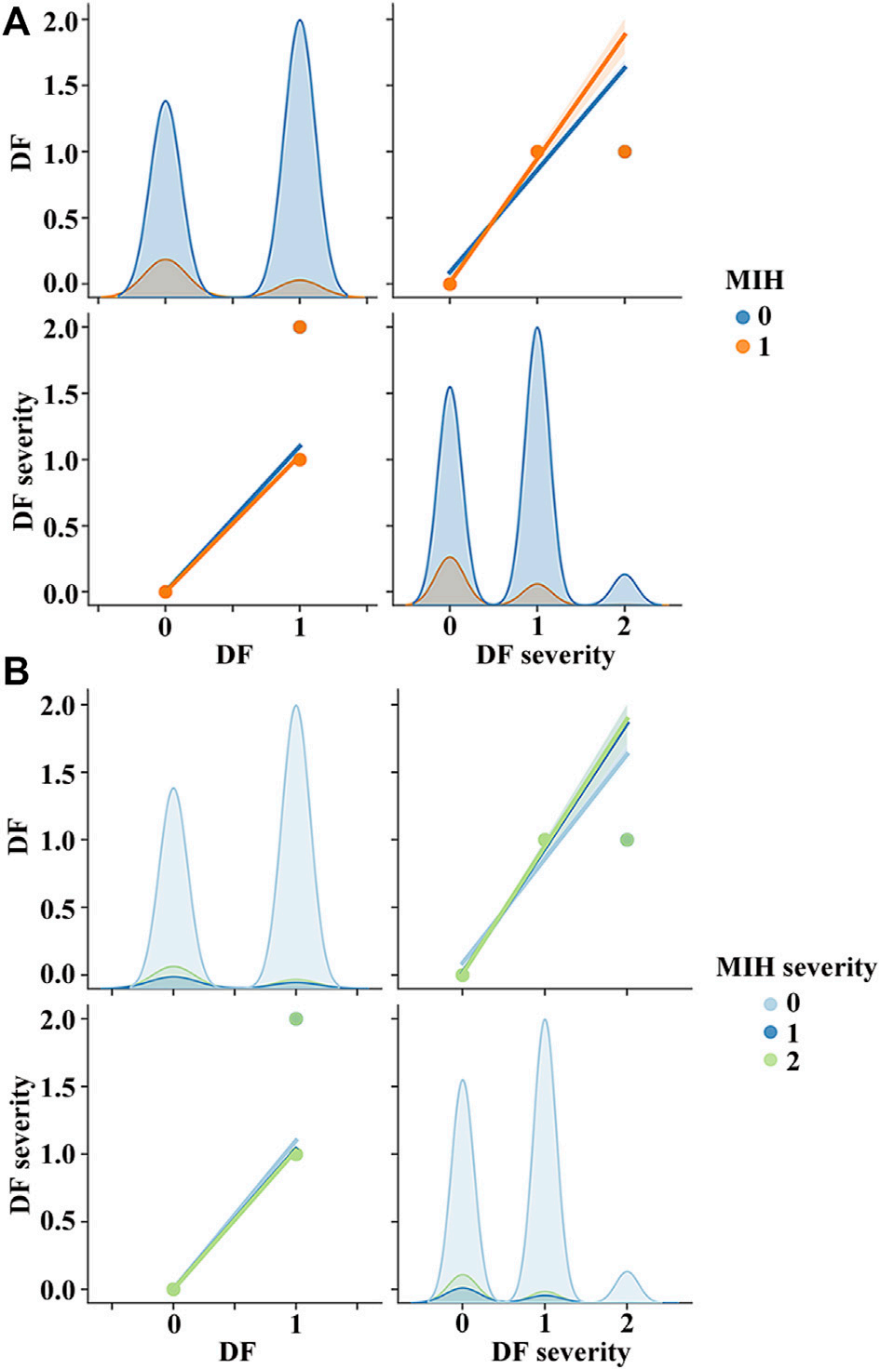
Pair plot was used to show relations among MIH, MIH severity, DF, and DF severity. During data preprocessing, the diagnoses of MIH or non-MIH, as well as DF or non-DF, were encoded using a binary encoder as 0 and 1. Thus, MIH or DF became 1, and non-MIH or non-DF became 0. Depending on the severity of MIH or DF, the level of MIH or DF was classed as normal (0), mild (1), or severe (2). **(A)** MIH was strongly negatively correlated with DF and DF severity. **(B)** Severity of MIH was reduced, followed by the incidence of DF and the strength of DF severity.

To find the relationship between MIH and DF, we used a pair plot. In Python, the pair plot showed that MIH (Figure 6A) was strongly negatively correlated with DF and DF severity. The severity of MIH was reduced, followed by the incidence of DF and the strength of DF severity (Figure 6B).

## 4 Discussion

In this study, we used data from 1,568 children to develop machine learning-based risk scores to predict the risk for MIH in an endemic fluorosis region in central China. Lankao is a typical quality-induced water shortage area, where there are various underground waters of poor quality, such as saltwater, brackish water, and high-hardness water. However, some regions have normal-quality groundwater that can be drunk directly. All participants were born and raised locally, and the natural groundwater source was the only drinking water before 2015. We have certitude that local shallow groundwater affected the permanent tooth mineralization of the recruited children.

MIH prevalence values reported by different studies are heterogeneous, varying from 2.4% to 44% in different areas (Dietrich et al., 2003; Calderara et al., 2005; Jasulaityte et al., 2007; Preusser et al., 2007; Ng et al., 2015) and from 2.8% to 25.5% in China (Cho et al., 2008; Sui et al., 2017; Li and Li, 2012; Zhang et al., 2020, Zhang et al.). The prevalence in this study was 13.7%, similar to the estimated world average of 12.9% (Schwendicke et al., 2018). Although there was a higher proportion of female patients with MIH, which is in agreement with a previous study (Mejare et al., 2005), the difference was not statistically significant. Spearman correlation analysis also indicated that sex does not seem to be a determining factor.

We qualified MIH as mild or severe to evaluate its severity, according to the criteria described by Mathu-Muju and Wright (2006). MIH is most often encountered in a mild form (Garcia-Margarit et al., 2014; Mittal and Sharma, 2015), which is consistent with the results of this study, in which 59.1% of the MIH cases were mild and 40.9% were severe at the tooth level (Table 2).

Recent developments in MIH research have focused on prevalence, and there are few prediction studies of early occurrence. In this study, a supervised machine learning model was constructed by incorporating a variety of factors that impact the occurrence of MIH in endemic fluorosis regions. The nomograms showed that sex had no influence on occurrence, which is consistent with previous studies (Yi et al., 2021; Sosa-Soto et al., 2022). Exposure to low-quality shallow underground water did not increase the likelihood of developing MIH. Significantly, non-DF had a substantial influence on the early occurrence of MIH, and the more severe the DF, the lower the occurrence.

A definitive conclusion has not been reached regarding the association between MIH prevalence and dental fluorosis. Thus, we explored the relationship between MIH and DF in children living in this endemic fluorosis region. As expected, the prevalence of fluorosis was as high as 54.8%. Spearman correlation analysis showed that MIH was negatively correlated with DF and DF severity; logistic regression analysis showed that DF had a protective correlation with MIH and that the effect of protection became more obvious with increasing severity. This finding accords with previous studies, showing that the prevalence of MIH was lower in the fluoridated area (10.8%) than in all non-fluoridated areas combined (17.35%) (Balmer et al., 2012). At the surface level, MIH frequency was lower in the presence of DF (Restrepo et al., 2022). Poisson regression analysis in a previous study showed that the *p*-value of the association between dental fluorosis and MIH was 0.084 and the OR ratio 0.63 (95% CI: 0.37–1.06) (Fernandes et al., 2021), which is similar to our findings. Enamel in patients diagnosed with MIH and fluorosis may not have completed maturation during amelogenesis (Malmberg et al., 2019; Fernandes et al., 2021). One possible explanation is that the affected teeth that initially erupt are hypomineralized; however, relatively long-term exposure to optimum levels of fluoride encourages remineralization. Finally, this continued remineralization could reduce or even change the defective clinical appearance.

According to the Fourth National Oral Health Survey in 2005, the caries prevalence in permanent teeth in China was 38.5% for the 12-year age group (Chen et al., 2018; Quan et al., 2018). The caries activity in Lankao City was low (14.2% of all teeth and 5.6% of only permanent teeth), which indicated that the high fluoride concentration of local water endowments truly prevented dental caries. In the nomograms, children with defective enamel were more prone to experiencing caries; logistic regression analysis showed that dental caries were positively correlated with MIH (OR = 1.843; 95% CI: 1.260–2.694). Most studies have shown a relationship between increased dental caries and children with MIH compared to those without MIH (Heitmüller et al., 2013; Petrou et al., 2015; Kosma et al., 2016). Furthermore, severe MIH cases had significantly higher caries prevalence than those with mild MIH (91.7% and 68.7%, respectively, *p* < 0.01; data not shown), suggesting that severe MIH increases the likelihood of caries in hypomineralized teeth. Teeth affected by MIH present a porous enamel surface due to poor mineral quality, which increases the likelihood of developing dental caries.

Unexpectedly, the prevalence of poor oral hygiene reached 91.5% in this region (Table 1), which increased the incidence of caries and worsened the severity of lesions. The nomogram showed that oral hygiene status did not affect the occurrence of MIH, and Spearman correlation analysis also found no relationship between oral hygiene status and MIH or MIH severity.

Altogether, the present study constructed a supervised machine learning algorithm to predict the occurrence of MIH in an endemic fluorosis region in central China, and the nomograph showed that MIH occurrence decreased with increasing DF severity. Then, we examined the association between MIH and DF and found a negative relationship, suggesting that DF should be considered a protective factor within the multifactorial etiology of MIH Zang et al., 2019.

## Data Availability

The original contributions presented in the study are included in the article/supplementary material; further inquiries can be directed to the corresponding author.
